# Are the Effects of the Cholera Toxin and Isoproterenol on Human Keratinocytes’ Proliferative Potential Dependent on Whether They Are Co-Cultured with Human or Murine Fibroblast Feeder Layers?

**DOI:** 10.3390/ijms19082174

**Published:** 2018-07-25

**Authors:** Sergio Cortez Ghio, Laurence Cantin-Warren, Rina Guignard, Danielle Larouche, Lucie Germain

**Affiliations:** 1 Université Laval Research Center on Experimental Organogenesis/LOEX, Québec, QC G1J 1Z4, Canada; sergio.cortez-ghio.1@ulaval.ca (S.C.G.); laurence.cwarren@hotmail.ca (L.C.-W.); rina.guignard@crchudequebec.ulaval.ca (R.G.); Danielle.larouche@crchudequebec.ulaval.ca (D.L.); 2 Regenerative Medicine Division, CHU de Québec—Université Laval Research Centre, Québec, QC G1J 1Z4, Canada; 3 Department of Surgery, Faculty of Medicine, Université Laval, Québec, QC G1V 0A6, Canada

**Keywords:** keratinocyte, feeder layer, cholera toxin, isoproterenol, cell culture, statistical model

## Abstract

Human keratinocyte culture has provided the means to treat burns, wounds and skin pathologies. To date, to efficiently culture keratinocytes, cells are cultured on an irradiated feeder layer (iFL), either comprising human (iHFL) or murine (i3T3FL) fibroblasts, and the culture medium is supplemented with a cyclic adenosine monophosphate (cAMP) accumulation inducing agent such as isoproterenol (ISO) or cholera toxin (CT). Previous studies have characterized how the feeder layer type and the cAMP inducer type influence epithelial cells’ phenotype independently from one another, but it is still unknown if an optimal combination of feeder layer and cAMP inducer types exists. We used sophisticated statistical models to search for a synergetic effect of feeder layer and cAMP inducer types on human keratinocytes’ proliferative potential. Our data suggests that, when culturing human keratinocytes, using iHFL over i3T3FL increases population doublings and colony-forming efficiency through signaling pathways involving Ak mouse strain thymoma (Akt, also known as protein kinase B) isoforms 1 to 3, signal transducer and activator of transcription 5 (STAT5), p53, and adenosine monophosphate activated protein kinase α1 (AMPKα1). Both tested cAMP inducers ISO and CT yielded comparable outcomes. However, no significant synergy between feeder layer and cAMP inducer types was detected. We conclude that, to promote human keratinocyte growth in the early passages of culture, co-culturing them with a human feeder layer is preferable to a murine feeder layer.

## 1. Introduction

Massive expansion of human keratinocytes in vitro was developed by Rheinwald and Green in 1975 [1]. Nowadays, cultured epithelial cells are used as a model system to study cellular and molecular interactions or mechanisms, and to investigate various skin pathologies [2,3,4]. Because this system enables epithelial stem cell retention, autologous keratinocytes can be grafted on patients as cell suspensions or epithelial sheets, thus providing means to treat superficial burns or to facilitate donor site regeneration [5,6,7,8]. For severe burns and chronic ulcers, cultured bi-layered skin substitutes—containing both dermal and epidermal cells—can also be grafted directly onto affected areas to promote healing [9,10,11,12,13]. Epithelial stem cell retention is however highly dependent upon culture conditions.

The system originally proposed by Rheinwald and Green [1] included culturing keratinocytes on murine lethally irradiated embryonic fibroblast (i3T3) feeder layers (i3T3FL). It also entailed supplementing the culture medium with cholera toxin (CT), a cyclic adenosine monophosphate (cAMP) accumulation inducing agent. However, for clinical applications, both i3T3FL and CT present drawbacks. The i3T3 are xenogeneic cells and working with CT comprises harsh handling regulations because it might pose a risk to human health if certain concentration thresholds are exceeded. This has led to the use of alternative feeder layer and cAMP inducer types. For example, in clinical settings, i3T3FL have been substituted with irradiated human dermal fibroblast feeder layers (iHFL), which have been shown to yield phenotypically-comparable keratinocyte cultures [14,15,16,17,18]. Indeed, it has been shown that human keratinocytes co-cultured with either iHFL or i3T3 display similar growth rates [15,17], clonogenicity [14], morphology [15] and viability [16]. In regards to cAMP inducers, isoproterenol (ISO), a clinically approved synthetic chemical compound with well-established effects in humans, can be used to replace CT. However, ISO and CT relative effectiveness has not reached a consensus. It has been reported that CT-supplemented medium allows for a higher colony-forming efficiency over ISO-supplemented medium in human keratinocytes [19]. It has also been shown that culturing canine oral mucosal epithelial cells with either ISO or CT does not significantly affect proliferative potential nor colony-forming efficiency [20]. Additionally, CT slightly outperforms ISO in terms of colony-forming efficiency when culturing human oral mucosal cells [20]. Lastly, another team described an enhanced proliferative potential and overall smaller size of human corneal epithelial cells when they were cultured in a feeder-free system with ISO instead of CT [21].

Remarkably, even with well documented isolated effects of both feeder layer and cAMP inducer types on epithelial cells’ proliferative potential, no study has yet assessed if the effects of cAMP inducers (CT and ISO) on keratinocyte phenotype vary depending on which feeder layer type (i3T3FL or iHFL) the keratinocytes are co-cultured with. The aim of this study was therefore to determine if an optimal combination of feeder layer type and cAMP inducer type exists. To achieve this, we used aligned rank transform linear mixed models in a balanced multifactorial design to evaluate the isolated and combined effects of the feeder layer type and the cAMP inducer type on different proliferative potential proxies. Additionally, we tested two grades of the cAMP inducer ISO (research- and clinical-grade). We observed that culturing human keratinocytes using iHFL over i3T3FL increased population doublings and colony-forming efficiency, and that Ak mouse strain thymoma (Akt, also known as protein kinase B), signal transducer and activator of transcription 5 (STAT5), p53, and adenosine monophosphate activated protein kinase α1 (AMPKα1) signaling pathways differential activation may be involved in these discrepancies. Both cAMP inducers tested in this article (ISO and CT) yielded comparable outcomes.

## 2. Results and Discussion

### 2.1. An Optimal Combination of Feeder Layer Type and of cAMP Inducer Type on Cultured Human Keratinocytes’ Proliferative Potential Was Not Detected in the Early Passages

The main objective of this study was to determine if an optimal combination—in terms of proliferative potential—of the types of feeder layer and cAMP inducer exists when culturing human keratinocytes, as it is established that both can influence the phenotype of cultured epithelial cells [14,15,16,17,19,20,21]. To achieve this, we used conservative sophisticated statistical models adjusted with factors that could potentially affect keratinocytes’ proliferative potential (cryopreservation, anatomical site and passage number). We chose to define keratinocytes’ proliferative potential as the combination of three cell growth-related attributes: their daily population doublings, their mean population cell size, and their colony-forming efficiency. These variables are adequate proxies in appraising keratinocytes’ proliferative potential, as together they allow for efficient estimations of growth rate, commitment into differentiation, and clonogenicity. Indeed, when cultured keratinocytes undergo differentiation, proliferation is halted, and cells enlarge. Smaller keratinocytes thus tend to proliferate more in comparison with their larger counterparts [22]. Additionally, colony-forming efficiency translate into the capacity to adhere and to produce progeny [22,23]. Calculating colony-forming efficiency in successive passages also allows for an estimation of stem cell retention through culture. In this experiment, only large colonies (holoclones) were considered, as colony size is indicative of how differentiated the original cell of a given colony initially was. Holoclones are derived from stem cells, as opposed to meroclones and paraclones, which are derived from transit-amplifying and differentiated cells respectively [23].

**Figure 1:**
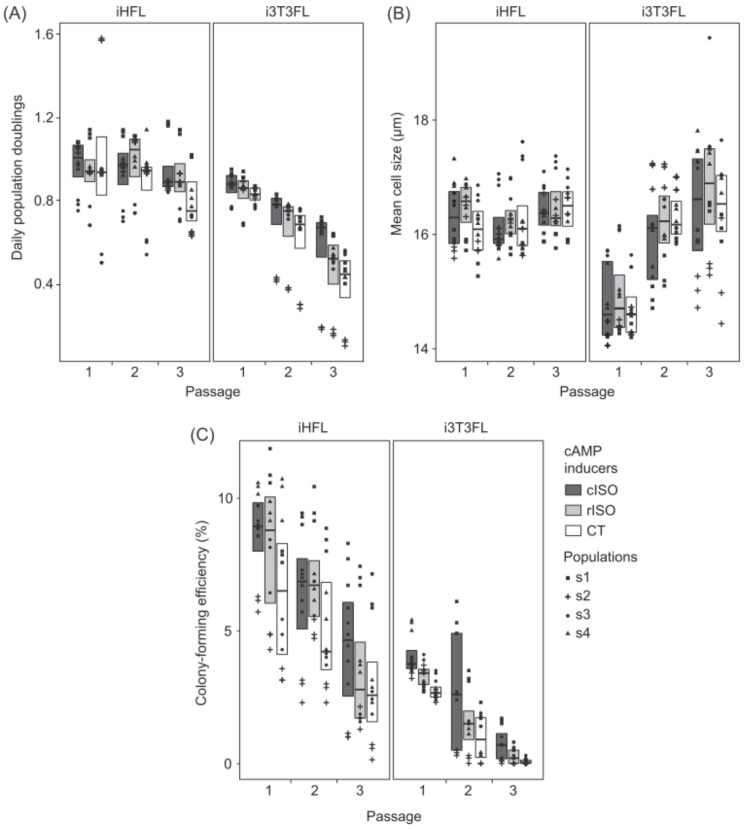
Box plots illustrating differences in (**A**) daily population doublings, (**B**) mean population cell size, and (**C**) colony-forming efficiency. Box plots show Q1 (quartile 1), median value, and Q3 (quartile 3). Triplicate values are shown as an overlaying dot plot. Keratinocyte populations (s1 to s4) are identified with symbols (s1: square, s2: cross, s3: circle, and s4: triangle). Box plot fill colors show what cAMP inducers were used (cISO: dark gray, rISO: light gray, and CT: white). Left panels show human keratinocytes co-cultured with irradiated human dermal fibroblast feeder layer (iHFL) and right panels those co-cultured with irradiated Swiss murine embryonic fibroblast feeder layer (i3T3FL).

We did not detect significant statistical interaction effects of the feeder layer type and of the cAMP inducer type on keratinocytes’ daily population doublings, mean population cell size, and colony-forming efficiency (Figure 1). This suggests that, in the early passages of culture, whether feeder layers comprise human or murine fibroblasts does not influence the effects of ISO and CT on cultured human keratinocytes’ proliferative potential.

It is likely, however, that the interpretation of these results cannot be extended to later passages. For this study, we restricted our experiments to the first 3 passages after primoculture (P1, P2, and P3) because we were mainly interested in the translational value of our findings on our culture protocols. It is known that as passages advance, keratinocytes can suffer chromosomal rearrangements [24] and a reduction of telomerase activity which leads to telomere shortening [25]. Therefore, it is generally preferred to use P1 to P3 keratinocytes when producing bio-engineered skin equivalents for clinical purposes [5,6,26]. Nevertheless, conducting these experiments into later passages could have yielded different results, as it has been shown that keratinocytes’ mean size, growth rate and key molecular processes can vary in later culture passages [15,18,27].

### 2.2. Co-Culturing Human Keratinocytes with iHFL Rather Than i3T3FL Results in an Increased Proliferative Potential in the Early Passages

Our results indicate that co-culturing human keratinocytes with iHFL rather than i3T3FL yields a higher growth rate and increased clonogenicity. Indeed, co-culturing keratinocytes with iHFL rather than i3T3FL resulted in a 40.9% increase in daily population doublings (Figure 1A and Table 1) and a 3.2-fold rise in colony-forming efficiency (Figure 1C and Table 1). Intriguingly enough, keratinocytes’ mean size was increased by 2.9% when cells were co-cultured with iHFL instead of i3T3FL (Figure 1B and Table 1). These effects were independent of which cAMP inducer type was supplemented into the medium and of the number of passages keratinocytes were cultured for.

As mentioned earlier, the increased growth rate and clonogenicity are typically associated with a less advanced state of differentiation, and less differentiated cells are generally smaller than their more differentiated counterparts [22]. It is therefore surprising that keratinocytes co-cultured with iHFL presented increased daily population doublings and colony-forming efficiency but were also slightly larger than those cultured on i3T3FL. This discrepancy could be explained by the fact that irradiated human dermal fibroblasts are larger in size than irradiated Swiss murine embryonic fibroblasts and that fibroblasts remain present in the cultures (especially in regions uncolonized by keratinocytes; See Supplementary Materials Table S1) and are, thus, resuspended with keratinocytes after trypsination. They might therefore lightly bias the automated cell measurements. Nevertheless, the biological effect of such a minor size difference (2.9%) might be inconsequential when massively expanding human keratinocytes in culture considering the much larger effects of iHFL on growth rate and clonogenicity.

Other studies did not find human keratinocytes’ phenotype to be altered when substituting the conventional i3T3FL for iHFL [14,15,16,17]. The differences between our observations and what the literature reports can however be partially explained. First, it is conceivable that the power of our statistical models has made it possible to detect differences between iHFL and i3T3FL co-cultures that could not have been detected otherwise. Second, previous studies employed different methodological approaches than us. Contrary to us, Bisson et al., cultured and measured keratinocytes’ growth and size until they reached senescence (P18) [15]. They also did not compare the effects of both feeder layer types on keratinocytes prior to P4, thus excluding observations on P1, P2, and P3 [15], the passages on which we focused herein. Auxenfans et al., like us, did use early passage keratinocytes (P2 and P3) for their colony-forming efficiency assays, but used irradiated fibroblasts from a 9 year-old patient as their feeder layer instead of neonate cells [14], and feeder layer donor-specific performance differences have been reported in the past [15]. Jubin et al., showed that irradiated human and murine fibroblasts were equally able to support keratinocyte expansion in co-culture for over 3 weeks of serial expansion [17]. However, they seeded irradiated human fibroblasts simultaneously with keratinocytes instead of several days earlier [17], possibly dampening the conditioning effect of the fibroblasts on the medium. Finally, Bullock et al., found no differences between iHFL and i3T3FL co-cultures on P1 keratinocytes’ viability [16]. Although, observing keratinocytes on more than a single passage is of the utmost importance, because we observed in this study that the number of passages in culture exacerbated the differences between iHFL and i3T3FL.

**Figure 2:**
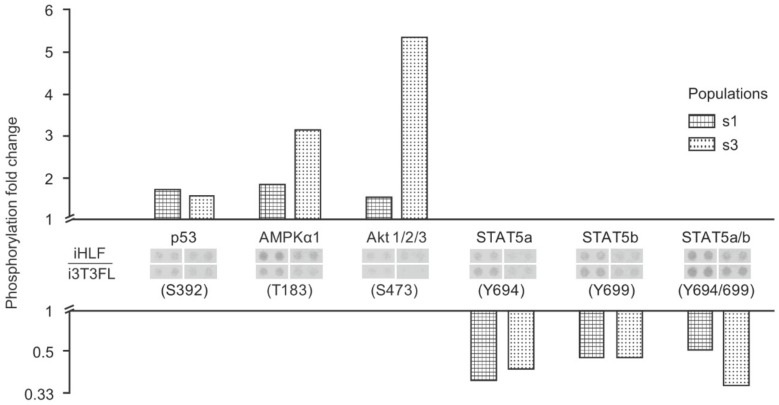
Bar plot illustrating relative differences in key kinase phosphorylation levels for two keratinocyte populations co-cultured either with iHFL or i3T3FL (iHFL/i3T3FL ratio). Keratinocytes used in this analysis were at P2 and were cultured in rISO-supplemented medium. Keratinocyte populations (s1 and s3) are identified with pattern fills (s1: squares and s3: circles). Only kinases which were at least 1.5-fold differentially phosphorylated between conditions for both populations are shown. Membrane blots are shown in between their respective kinase ID and phosphorylated site.

Indeed, we detected significant statistical interaction effects of the feeder layer type used and the number of passages the keratinocytes were cultured for on daily population doublings and on mean population cell size. The difference in daily population doublings between keratinocytes co-cultured with either iHFL or i3T3FL increased by 35.5% between P1 and P2 (Figure 1A and Table 1), and the difference in cell size between keratinocytes co-cultured with either iHFL or i3T3FL decreased 21-fold after P1 (Figure 2B and Table 1), meaning that keratinocytes co-cultured with i3T3FL decreased in daily population doublings and increased in mean size significantly faster than those co-cultured with iHFL. These results strengthen the argument in favor of iHFL over i3T3FL for keratinocyte proliferation, accentuate the importance of conducting experiments on multiple passages, and suggest that these trends might extend beyond P3.

### 2.3. Culturing Human Keratinocytes with Either ISO- or CT-Supplemented Medium Does Not Significantly Influence Proliferative Potential in the Early Passages

In a similar fashion to a previous study conducted on cultured canine oral epithelial cells [20], our data show that using either ISO- or CT-supplemented medium does not significantly affect cells’ daily population doublings or mean population cell size in the early passages. This lack of effect was independent of which feeder layer type keratinocytes were co-cultured with and of the number of passages they were cultured for (Figure 1A,B). These results are intriguing as CT-supplemented medium has been shown to yield an increased colony-forming efficiency when compared with ISO-supplemented medium on both human skin keratinocytes [19] and human oral mucosal epithelial cells [20]. Furthermore, Green compared four distinct cAMP inducing agents (CT, ISO, dibutyryl cAMP, methyl isobutyl xanthine) on human keratinocyte cultures in 1978, and CT outperformed them all in terms of colony-forming efficiency and colony size [19]. ISO came close second and contrary to CT, its effectiveness in promoting keratinocyte clonogenicity seemed to be concentration-specific. However, only one neonate-isolated keratinocyte population was investigated in this study. Conversely, ISO has been shown to yield an increased growth rate and a smaller mean cell size on human corneal epithelial cells when compared with CT [21]. It might thus be possible that the influence of the cAMP inducer type on epithelial cell cultures is species- and tissue-specific.

Nonetheless, based on our observations, we endorse the use of ISO- over CT-supplemented medium for the culture of early-passage human keratinocytes. ISO is a synthetic, well characterized, and safe molecule that is available in GMP (good manufacturing practice) grade. It thus requires far less regulatory approbations than CT (due to its association to risks to human health at high concentrations). Another interesting point brought forward by Green in favor of ISO is that its effects on keratinocytes are reversible through medium changes as opposed to those of CT [19].

### 2.4. Culturing Human Keratinocytes with Either cISO- or rISO-Supplemented Medium Does Not Significantly Influence Proliferative Potential in the Early Passages

Currently available clinical-grade ISO (cISO; GMP grade) comes in single-use containers that cannot be stored for later use. It is also more expensive than CT (1.90 $ CAN/L of culture medium to 0.97 $ CAN/L of culture medium). We thus decided to verify if the research-grade powdered equivalent (rISO), which is storable at −80 °C for several months and is much cheaper (1.65 $ CAN/1000 L of culture medium) than both cISO (1148.5-fold) and CT (585.5-fold), is a viable alternative for research and development purposes. Our results indicate that the effects of cISO and rISO supplementation on cultured human keratinocytes did not significantly differ from one another in the early passages (Figure 1). Nevertheless, colony-forming efficiency was increased by 1.49% when the culture medium contained cISO rather than CT, but there were no significant differences between rISO and CT, and between rISO and cISO (Figure 1C and Table 1). This was observed independently of which feeder layer type keratinocytes were co-cultured with and of the number of passages they were cultured for. Note that cISO’s minor outperformance of CT was the only difference between cISO and rISO.

To the best of our knowledge, there are no other reported comparisons for ISO grades on epithelial cells’ proliferative potential in the literature. Considering our data, we recommend using rISO rather than cISO for research and development purposes because keratinocytes cultured with either rISO or cISO display comparable phenotypes, and because rISO is considerably more affordable than cISO.

### 2.5. The Increased Growth Rate and Clonogenicity Observed When Keratinocytes Are Co-Cultured with iHFL Rather Than i3T3FL Could Be Explained by Differential Activation of Key Signaling Pathways

In this study, we found that co-culturing keratinocytes with iHFL rather than i3T3FL resulted in an increase in growth rate and clonogenicity. To investigate what cellular mechanisms could be involved in these feeder layer-related differences, we performed a kinase phosphorylation profile analysis with whole cell protein extracts from two keratinocyte populations at P2 which were cultured in rISO-supplemented medium.

We showed herein that some kinases were differentially phosphorylated between keratinocytes co-cultured with either feeder layer type; while p53, AMPKα1, and Akt isoforms 1 to 3 displayed an increase in their phosphorylation status when keratinocytes were co-cultured with iHFL rather than i3T3FL, STAT5 proteins were conversely more phosphorylated when cells were co-cultured with i3T3FL rather than iHFL (Figure 2). These results are consistent with the growth rate and clonogenicity differences we observed between keratinocytes co-cultured with either iHFL or i3T3FL. Indeed, Akt isoforms are signaling proteins that promote cell proliferation and survival through the activation of the mTOR (mechanistic target or rapamycin) complex [28], and STAT5 proteins are transcription factors that mediate cell cycle progression and apoptosis in response to various cytokines [29,30]. Akt isoforms and STAT5 phosphorylation profiles observed in this study are also in line with one of our group’s recent publication on an in vitro reconstructed corneal epithelium wound healing model. Couture et al. found that STAT5a and STAT5b were less phosphorylated in post-wound regenerating neo-epithelium cells as opposed to less actively proliferating cells of unwounded regions of the model [31]. In that same study, and in accordance with our results, p53 and Akt isoforms 1 to 3 displayed increased phosphorylation statuses in neo-epithelium cells when compared to those of unwounded regions [31].

### 2.6. Perspectives

Future studies should substantiate these findings by culturing keratinocytes beyond P3; possibly even until senescence is reached. The herein used proliferative potential proxies should also be complemented with molecular biomarker expression analyses [15,17,18,32,33,34]. In light of our results, further characterization of the STAT-, p53-, AMPKα1-, and Akt-related signaling pathways in skin epithelial cell cultures is also needed. Additionally, investigating proteins implicated in structure (keratin 1, 5, 10 and 14) and adhesion (e.g., integrins α3, α6, and β4, laminins α3 and γ3) growth regulation (e.g., sp1, and TERT), and differentiation regulation (MAP3K12, filaggrin, involucrin, transglutaminase, and loricrin) may notably provide us with a clearer understanding of the mechanisms by which fibroblast feeder layers contribute to sustain the proliferative potential of cultured human keratinocytes.

## 3. Materials and Methods

### 3.1. Ethical Considerations

This study was conducted in accordance with our institution’s guidelines and the Declaration of Helsinki. This study was approved (no. 2012-1251; 1992/04/28) by our institution’s committee for the protection of human subjects (comité d’éthique de la recherche du CHU de Québec—Université Laval). Tissue donors gave their written informed consent for the use of retrieved tissues for research or educational purposes.

### 3.2. Cell Populations

All human keratinocyte populations studied (designated s1 to s4) were obtained from healthy subjects undergoing esthetic elective surgery. Keratinocytes were isolated from the excess skin removed either after breast-reduction (s1 and s3) or face-lift (s2 and s4) surgeries from 26-(s1), 65-(s3), 67-(s2) and 62-years-old (s4) female subjects. Keratinocytes were either isolated and immediately cultured (s3 and s4) or were cryopreserved for 14 and 11 years after they were primocultured (s1 and s2 respectively) prior to their use in this study. Cells from different donors were never pooled. Table A1 in Appendix A displays all four keratinocyte populations studied for this article and summarizes their characteristics.

### 3.3. cAMP Accumulation Inducing Agents

Complete keratinocyte culture medium (ckDME-Ham; See Appendix B) was supplemented with either 10^−10^ M CT (Sigma-Aldrich, St. Louis, MO, USA) or 10^−6^ M ISO (2.12 × 10^−5^ mg/mL). Two different ISO grades were tested: a clinical-grade single-use ISO (cISO; Sandoz, Boucherville, QC, Canada) and a research-grade powdered ISO (rISO; Sigma-Aldrich) diluted in 0.0005% *v*/*v* 2N HCl (Thermo Fisher Scientific, Ottawa, ON, Canada).

### 3.4. Feeder Layers

The iHFL are cultured irradiated human neonate foreskin dermal fibroblasts [15]. Prior to this study, our laboratory had established a cryobank (See Appendix B) of these pre-irradiated (6000 rad) cells. Irradiated fibroblasts were thawed and plated at 2 × 10^5^ cells/25 cm^2^ in either ISO- or CT-supplemented ckDME-Ham. This was done at least 7 (an up to 30) days before keratinocytes were seeded (to condition the culture medium) on top of them. The medium was changed every 7 days. If keratinocytes were to be seeded the same day a medium change was scheduled, only half of the medium was changed, so to keep it partly conditioned by the iHFL [14].

The 3T3 are a commercially available cultured Swiss murine embryonic fibroblast line [15]. These fibroblasts are cultured to confluence, passaged, and part of them are irradiated at 6000 rad weekly to maintain a steady supply of i3T3. For these experiments, i3T3 were trypsinized (See Appendix B) and seeded simultaneously with keratinocytes at 5 × 10^5^ cells/25 cm^2^ in ckDME-Ham.

### 3.5. Keratinocyte Isolation

Skin specimens were transported to the lab in transport medium (See Appendix B) on the same day the surgeries took place and were thoroughly washed (See Appendix B) on arrival. Samples were subsequently cut into small strips (3 mm × 100 to 150 mm) and incubated overnight at 4 °C in a thermolysin [35] dissociation solution (See Appendix B). The next day, the epidermis was peeled from the dermis with tweezers. The small epidermal strips were then incubated 30 min at 37 °C in the trypsin/EDTA solution on a stirring plate. Trypsin activity was neutralized by doubling the volume of the suspension with CT-supplemented (s1 and s2) or cAMP inducer-free (s3 and s4) ckDME-Ham. Note that s1 and s2 were isolated and primocultured over 10 years prior to these experiments (see Section 3.2), and that keratinocytes were then routinely cultured on i3T3FL with CT-supplemented ckDME-Ham. After trypsin inactivation, cells were centrifuged at 300× *g* for 10 min and resuspended in either CT-supplemented ckDME-Ham (s1 and s2) or cAMP inducer-free ckDME-Ham (s3 and s4).

### 3.6. Keratinocyte Primoculture (P0)

Keratinocyte populations s1 and s2 (isolated over 10 years prior to this study) were seeded at 7 × 10^5^ cells/25 cm^2^ on i3T3FL in CT-supplemented ckDME-Ham. Keratinocyte populations s3 and s4 were seeded at densities of 3.5 × 10^5^ cells/25 cm^2^ (on iHFL) or 7 × 10^5^ cells/25 cm^2^ (on i3T3FL) in either ISO- or CT-supplemented ckDME-Ham. Keratinocytes were kept at 37 °C, 8% CO_2_, and 95 ± 5% humidity. Medium was changed 3 times per week. Cells were either cryopreserved (s1 and s2) or passaged (s3 and s4) when P0 reached a near-confluent state (70–95%).

### 3.7. Keratinocyte Subculture

From P1 forward, keratinocytes were plated in triplicate to reduce handling-related variance. Keratinocytes were plated in 25 cm^2^ culture flasks (Thermo Fisher Scientific) and passaged with a trypsin/EDTA solution generally when cultures reached 70–95% confluence or when more than seven days had gone by since seeding (see Supplementary Materials Table S1). Cultures were not allowed to reach confluence. Trypsin activity was inhibited by doubling the volume of the suspension with cAMP inducer-free ckDME-Ham. Cells were then centrifuged and resuspended in either ISO- or CT-supplemented ckDME-Ham. Seeding density for P1 to P3 ranged between 1 × 10^5^ cells/25 cm^2^ and 3 × 10^5^ cells/25 cm^2^ (see Supplementary Materials Table S1). Keratinocytes were maintained in culture and passaged until P3.

### 3.8. Keratinocyte Cryopreservation and Thawing

Keratinocyte populations s1 and s2 were isolated and primocultured until they reached 70–95% confluence. Keratinocytes were then trypsinized and resuspended at 2 × 10^6^ cells/mL (s1) and 4 × 10^6^ cells/mL (s2) in cryopreservation medium (See Appendix B). Cryovials (Thermo Fisher Scientific) containing 1 mL aliquots of the cell suspensions were kept on ice during handling and were rapidly transferred into a freezing container (Thermo Fisher Scientific) filled with 4 °C 100% isopropyl alcohol (Commercial Alcohols, Boucherville, QC, Canada). The container was then placed at −80 °C overnight and cryovials were transferred into liquid nitrogen. Keratinocytes were kept frozen in liquid nitrogen for 14 (s1) and 11 (s2) years prior to this study.

Keratinocyte populations s1 and s2 were thawed by placing the cryovials in a 37 °C water bath for no more than 1 min. The cell suspensions were then transferred into 4 °C cAMP inducer-free ckDME-Ham and centrifuged at 300× *g* for 10 min. Keratinocytes were then resuspended in cAMP inducer-free ckDME-Ham and seeded in P1. Mortality rates were estimated with trypan blue (Sigma-Aldrich) staining and a hemacytometer under a phase-contrast microscope (Olympus, Toronto, ON, Canada). Mortality never exceeded 7%.

### 3.9. Keratinocytes’ Proliferative Potential Proxies

Keratinocytes’ daily doublings (*Ddoubs*) were calculated with the following formulae:

(1) $$Ndoubs=\frac{\log(Ktryp/Kseed)}{\log\left(2\right)}$$

(2) $$Ddoubs=\frac{Ndoubs}{D}$$

where *Ktryp* is the number of keratinocytes trypsinized at the end of a given passage (measured with a Beckman Coulter automated cell counter), *Kseed* is the number of keratinocytes seeded at the beginning of the passage, *Ndoubs* is the total number of population doublings that occurred during the passage, and *D* is the lenght of the passage in days (from seeding to trypsination, rounded to the quarter hour; see Supplementary Materials Table S1).

Populations’ mean cell sizes were also measured with the automated cell counter when keratinocytes were trypsinized at the end of a passage.

Keratinocyte colony-forming efficiency assays were performed over 13 days in 25 cm^2^ culture flasks. Medium was changed at days 4 and 10. Colonies were fixed with 3.7% formol (ACP, Montreal, QC, Canada) and stained with a Nile blue A—rhodamine mixture (Sigma-Aldrich). Colony-forming efficiency percentages (*CFE*) were calculated with the following formulae:

(3) $$CFE=\frac{Nholo}{Kseed}\times 100$$

where *Nholo* is the number of colonies with a diameter larger than 4 mm, and *Kseed* is the number of keratinocytes seeded into the culture flask for the assay, which was either 700 when cells were seeded on iHFL or 1000 when cells were seeded on i3T3FL.

### 3.10. Kinase Phosphorylation Profile Assays

Each passage, unseeded suspended keratinocytes were washed in 1× PBS and centrifuged at 300× *g* for 10 min at 4 °C. Pellets were then dried and stored at −80 °C. Three years later, whole cell protein extracts we prepared by homogenizing keratinocyte frozen pellets from populations s1 and s3 (at P2, cultured in rISO-supplemented medium, and with both iHFL and i3T3FL) in lysis buffer (See Appendix B). Protein concentration was determined by the Bradford method. A human phospho-kinase antibody array kit (R&D Systems, Minneapolis, MN, USA) was then used accordingly to the manufacturer’s instructions. Equal amounts of cell lysates (300 μg) from each condition were incubated overnight with an array membrane, washed, and incubated with biotinylated detection antibodies. Signals were then detected with streptavidin-horseradish peroxidase and chemiluminescent detection reagents. Densitometric analyses of membrane blots were performed with Fiji (Image J, v1.52d) [36].

### 3.11. Statistical Analyses

All statistical analyses were carried out in R (v3.4.3, R Studio v1.1423) [37]. R-formatted data are available in the Supplementary Materials Table S2. An annotated script with the code used for every test conducted herein is also available in the Supplementary Materials Figure S1. Because initial parametric testing revealed that both the residuals’ normality and homoscedasticity assumptions were not met, a more conservative non-parametric approach was selected. A non-parametric three-way linear mixed model was fit for every proliferative potential proxy (response variables: population daily doublings, mean cell size, and colony-forming efficiency) using the ARTool (Aligned Rank Transform Tool) package (v0.10.4) [38]. The three categorical fixed factors against which models were fit were the feeder layer type (iHFL and i3T3FL), the cAMP inducer type (cISO, rISO, and CT), and the passage number (P1, P2, and P3). Two categorical random factors were also integrated into the models to correct for their possible influence over the response variables. These were the anatomical site populations were isolated from (face-lift tissue and breast-reduction tissue) and whether keratinocytes had previously been cryopreserved or not. When significant (*p* < 0.05) single-factor effects were detected by the Aligned Rank Transform models, Tukey post-hoc tests were performed with the lsmeans package (v2.27-61) [39] and significant (*p* < 0.05) pair-wise comparisons were reported. When significant (*p* < 0.05) factor interaction effects were detected by the Aligned Ranked Transform models, Holm-Bonferroni-adjusted chi-square post-hoc tests were performed with the phia package (v0.2-1) [40] and, again, significant (*p* < 0.05) pair-wise comparisons were reported. Note that the models were also fit with the keratinocyte population ID as the sole random factor (which corrects for every inherent quality of each cell population, like donor age or collection method). Effect sizes did not differ and *p*-value variations did not exceed 10%. The more conservative previously described models were thus selected.

## Figures and Tables

**Table 1:** Summary of significant (three-way align rank transform linear mixed models; *p* < 0.05) single factor effects and factor interaction effects on cultured human keratinocytes’ proliferative potential. Mean values are displayed.

^1^ Significant Factor Effects and Interactions	Keratinocytes’ Proliferative Potential Proxies		
	Daily Population Doublings	Population Mean Cell Size (μm)	Colony-Forming Efficiency (% of Holoclones)
Feeder layer type	iHFL > i3T3FL	iHFL > i3T3FL	iHFL > i3T3FL
	0.93 > 0.66	16.32 > 15.85	5.85 > 1.81
cAMP inducer type			cISO > CT
			4.56 > 3.07
Passage number	(P1 = P2) > P3	P1 < (P2 = P3)	P1 > P2 > P3
	(0.89 = 0.83) > 0.66	15.56 < (16.20 = 16.50)	5.58 > 4.01 > 2.00
^2^ Feeder layer type × passage number	iHFL − i3T3FL: P1 < (P2 = P3)	iHFL − i3T3FL: P1 > (P2 = P3)	
	0.11 < (0.31 = 0.38)	1.47 > (0.07 = −0.11)	

^1^ Only significant effects (*p* < 0.05) are reported in this table. ^2^ Passage-specific differences of the proxy means for keratinocytes cultured on iHFL when compared to keratinocytes cultured on i3T3FL.

## Supplementary Materials

Supplementary materials can be found at http://www.mdpi.com/1422-0067/19/8/2174/s1.

## Abbreviations

3T3	Swiss murine embryonic fibroblast cell line
Akt	Protein kinase B
AMPKα1	Adenosine monophosphate activated protein kinase α1
cAMP	Cyclic adenosine monophosphate
cISO	Clinical-grade ISO
ckDME-Ham	Complete keratinocyte culture medium (cAMP inducer-free)
CT	Cholera toxin
EDTA	Ethylenediaminetetraacetic acid
GMP	Good manufacturing practice
i3T3	Irradiated Swiss murine embryonic fibroblasts
i3T3FL	Irradiated Swiss murine embryonic fibroblast feeder layer
iFL	Irradiated feeder layer
iHLF	Irradiated human dermal fibroblast feeder layer
ISO	Isoproterenol
mTOR	Mechanistic target of rapamycin
PBS	Phosphate buffered saline
rISO	Research-grade ISO
STAT5	Signal transducer and activator of transcription 5

## Appendix A

**Table A1:** Keratinocyte populations used in this study.

Female Subjects (Age)	Anatomical Site	Cryopreservation
s1 (26 y/o)	Breast reduction tissue	* Cryopreserved
s2 (67 y/o)	Face-lift tissue	* Cryopreserved
s3 (65 y/o)	Breast reduction tissue	Never cryopreserved
s4 (62 y/o)	Face-lift tissue	Never cryopreserved

* s1 was cryopreserved for 14 years and s2 for 11 years prior to this study.

## Appendix B

### Media and Solutions

1. Complete keratinocyte culture medium (ckDME-Ham): Keratinocytes were cultured in three parts Dulbecco’s modified Eagle’s medium (DMEM; Thermo Fisher Scientific) and one part Ham’s F12 medium (Thermo Fisher Scientific) mixture with 5% *v*/*v* of inactivated FetalClone II serum (HyClone, Logan, UT, USA). Additionally, it contained 3.07 g/L of NaHCO_3_ (J.T. Baker, Phillipsburg, NJ, USA), 24.3 mg/L of adenine (Sigma-Aldrich), 5 μg/mL of insulin (Sigma-Aldrich), 10 ng/mL of epidermal growth factor (EGF; Austral Biologicals, San Ramon, CA, USA), 0.4 μg/mL of hydrocortisone (Calbiochem, La Jolla, CA, USA), 100 IU/mL of penicillin G (Sigma-Aldrich), 25 μg/mL of gentamicin (Galenova, Saint-Hyacinthe, QC, Canada), and either ISO or CT (see Section 3.4).
2. Cryopreservation medium: Keratinocyte populations s1 and s2, and irradiated foreskin dermal fibroblasts were cryopreserved in inactivated fetal calf serum (HyClone) containing 10% *v*/*v* of dimethyl sulfoxide (DMSO; Sigma-Aldrich).
3. Trypsin/EDTA solution: Keratinocytes and fibroblasts were detached from culture flasks and dissociated with a phosphate buffered saline (PBS) solution comprising 0.05% *w*/*v* of porcine trypsin 1–250 (Thermo Fisher Scientific), 0.01% *w*/*v* of ethylenediaminetretraacetic acid (EDTA; J.T. Baker), 2.8 mM of d-glucose (EMD Millipore, Burlington, MA, USA), 100 IU/mL of penicillin G (Sigma-Aldrich), 25 μg/mL of gentamicin (Galenova), and 0.00075% *w*/*v* of phenol red (J.T. Baker).
4. Transport medium: Tissue samples from elective surgeries were transported from the clinic to our lab in 10% *v*/*v* of fetal calf serum in high glucose (4.5 g/L) DMEM (containing sodium pyruvate and L-glutamine; Thermo Fisher Scientific) with 0.5 μg/mL of amphotericin B (Bristol-Myers Squibb, Saint-Laurent, QC, Canada), 100 IU/mL of penicillin G (Sigma-Aldrich), and 25 μg/mL of gentamicin (Galenova).
5. Washing solution: Tissue samples were washed in 1× PBS containing 0.5 μg/mL of amphotericin B (Bristol-Myers Squibb), 100 IU/mL of penicillin G (Sigma-Aldrich), and 25 μg/mL of gentamicin (Galenova).
6. Thermolysin dissociation solution: The epidermis and the dermis were dissociated after skin specimens were incubated in a 500 μg/mL of thermolysin (Sigma-Aldrich) in a 0.01 M 4-(2-hydroxyethyl)-1-piperazineethanesulfonic acid (HEPES; MP Biomedicals, Santa Ana, CA, USA) solution containing 0.67 mM of KCL (Thermo Fisher Scientific), 0.14 M of NaCl (Thermo Fisher Scientific), and 1 mM of CaCl_2_ (Sigma-Aldrich).
7. Lysis buffer: Whole cell protein extracts were prepared by homogenizing keratinocytes in a buffer containing 1% (*v*/*v*) Triton X-100 (Bio-Rad, Mississauga, ON, Canada), 50 mM Tris (pH 7.4; Bio Basic, Markham, ON, Canada), 150 mM NaCl (Thermo Fisher Scientific), 1 mM ethylenediaminetretraacetic acid (EDTA; J.T. Baker), 1 mM sodium orthovanadate (Sigma-Aldrich), and protease inhibitors (Roche Diagnostics, Laval, QC, Canada).
