# Supplementary material for: Are the Effects of the Cholera Toxin and Isoproterenol on Human Keratinocytes’ Proliferative Potential Dependent on Whether They Are Co-Cultured with Human or Murine Fibroblast Feeder Layers?
Source: Int J Mol Sci. 2018 Jul 25;19(8):2174. doi: 10.3390/ijms19082174 (PMC6121595; doi:10.3390/ijms19082174)

International Journal of Molecular Sciences

Supplementary Materials Table S1. Other culture-related information

Population	cAMP inducer type	Feeder layer type	Passage	Seeded Keratinocytes (1×10^5 cells/25 cm2)	Passage length (days)	Confluence reached (%)
s1	rISO	iHFL	P1	2	4.06	95
			P2	1	4.77	95
			P3	2	4.22	95
		i3T3FL	P1	1	7.08	95
			P2	1	6.83	95
			P3	1	6.92	90
	cISO	iHFL	P1	2	4.06	95
			P2	1	4.77	95
			P3	2	4.22	95
		i3T3FL	P1	1	7.08	95
			P2	1	6.83	95
			P3	1	6.92	90
	CT	iHFL	P1	2	4.06	95
			P2	1	4.77	85
			P3	2	4.22	90
		i3T3FL	P1	1	7.08	85
			P2	1	6.83	85
			P3	1	6.92	35
s2	rISO	iHFL	P1	2	4.04	85
			P2	2	3.77	95
			P3	2	4.21	85
		i3T3FL	P1	1	7.08	90
			P2	1	10.81	80
			P3	1	15.82	N/A
	cISO	iHFL	P1	2	4.06	90
			P2	1	4.77	90
			P3	2	4.22	90
		i3T3FL	P1	1	7.08	90
			P2	1	10.81	90
			P3	1	15.82	N/A
	CT	iHFL	P1	2	4.06	80
			P2	1	4.77	80
			P3	2	4.22	70
		i3T3FL	P1	1	7.08	80
			P2	1	10.81	40
			P3	1	15.82	N/A
s3	rISO	iHFL	P1	2	4.02	75
			P2	1	4.61	70
			P3	1	5.19	60
		i3T3FL	P1	2	6.02	95
			P2	1	5.93	75
			P3	1	7.16	45

	cISO	iHFL	P1	2	4.02	75
			P2	1	4.61	70
			P3	1	5.19	80
		i3T3FL	P1	2	6.02	95
			P2	1	5.93	85
			P3	1	7.16	90
	CT	iHFL	P1	2	4.02	65
			P2	1	4.61	60
			P3	1	5.19	60
		i3T3FL	P1	2	6.02	95
			P2	1	5.93	65
			P3	1	7.16	50
s4	rISO	iHFL	P1	1	5.11	85
			P2	1	4.89	85
			P3	2	4.14	80
		i3T3FL	P1	3	4.93	95
			P2	1	6.91	95
			P3	1	6.82	60
	cISO	iHFL	P1	1	5.11	90
			P2	1	4.89	95
			P3	2	4.14	75
		i3T3FL	P1	3	4.93	95
			P2	1	6.91	95
			P3	1	6.82	80
	CT	iHFL	P1	1	5.11	80
			P2	1	4.89	80
			P3	2	4.14	60
		i3T3FL	P1	3	4.93	95
			P2	1	6.91	75
			P3	1	6.82	40

Supplementary Materials Table S2. R-ready data

primo	anato	pop	camp	flayer	pass	xdd	xms	xhfe
cryo	breastred	s1	rISO	IHFL	P1	1.10	15.83	11.10
cryo	breastred	s1	rISO	IHFL	P2	1.16	15.81	9.67
cryo	breastred	s1	rISO	IHFL	P3	1.07	15.92	7.05
cryo	breastred	s1	rISO	i3T3FL	P1	0.91	14.32	2.87
cryo	breastred	s1	rISO	i3T3FL	P2	0.79	15.32	3.27
cryo	breastred	s1	rISO	i3T3FL	P3	0.64	16.49	0.63
cryo	breastred	s1	cISO	IHFL	P1	1.06	15.95	9.05
cryo	breastred	s1	cISO	IHFL	P2	1.18	15.82	9.24
cryo	breastred	s1	cISO	IHFL	P3	1.11	15.92	7.57
cryo	breastred	s1	cISO	i3T3FL	P1	0.92	14.23	3.77
cryo	breastred	s1	cISO	i3T3FL	P2	0.83	14.89	5.43
cryo	breastred	s1	cISO	i3T3FL	P3	0.72	15.95	1.60
cryo	breastred	s1	TC	IHFL	P1	0.93	15.68	7.81
cryo	breastred	s1	TC	IHFL	P2	1.00	15.75	8.43
cryo	breastred	s1	TC	IHFL	P3	0.96	15.89	6.33
cryo	breastred	s1	TC	i3T3FL	P1	0.85	14.31	2.57
cryo	breastred	s1	TC	i3T3FL	P2	0.73	15.95	1.80
cryo	breastred	s1	TC	i3T3FL	P3	0.46	16.11	0.03
cryo	facelift	s2	rISO	IHFL	P1	0.93	16.49	4.67
cryo	facelift	s2	rISO	IHFL	P2	1.15	16.24	5.00
cryo	facelift	s2	rISO	IHFL	P3	0.87	16.39	1.71
cryo	facelift	s2	rISO	i3T3FL	P1	0.86	14.43	3.53
cryo	facelift	s2	rISO	i3T3FL	P2	0.38	17.09	0.17
cryo	facelift	s2	rISO	i3T3FL	P3	0.17	15.40	0.00
cryo	facelift	s2	cISO	IHFL	P1	1.03	15.69	6.05
cryo	facelift	s2	cISO	IHFL	P2	1.01	15.98	2.81
cryo	facelift	s2	cISO	IHFL	P3	0.82	16.51	1.05
cryo	facelift	s2	cISO	i3T3FL	P1	0.88	14.26	3.43
cryo	facelift	s2	cISO	i3T3FL	P2	0.43	17.09	0.40
cryo	facelift	s2	cISO	i3T3FL	P3	0.19	15.00	0.07
cryo	facelift	s2	TC	IHFL	P1	1.55	15.77	3.29
cryo	facelift	s2	TC	IHFL	P2	0.98	15.85	2.71
cryo	facelift	s2	TC	IHFL	P3	0.61	16.55	0.48
cryo	facelift	s2	TC	i3T3FL	P1	0.82	14.42	2.60
cryo	facelift	s2	TC	i3T3FL	P2	0.30	16.94	0.00
cryo	facelift	s2	TC	i3T3FL	P3	0.12	15.24	0.00
nocryo	breastred	s3	rISO	IHFL	P1	0.71	16.80	7.67
nocryo	breastred	s3	rISO	IHFL	P2	0.82	16.86	6.43
nocryo	breastred	s3	rISO	IHFL	P3	0.70	17.27	1.71
nocryo	breastred	s3	rISO	i3T3FL	P1	0.68	16.11	3.83
nocryo	breastred	s3	rISO	i3T3FL	P2	0.74	16.58	1.53
nocryo	breastred	s3	rISO	i3T3FL	P3	0.48	18.17	0.33
nocryo	breastred	s3	cISO	IHFL	P1	0.77	16.60	9.00
nocryo	breastred	s3	cISO	IHFL	P2	0.78	16.96	6.29

nocryo	breastred	s3	cISO	IHFL	P3	0.84	16.92	5.33
nocryo	breastred	s3	cISO	i3T3FL	P1	0.76	15.69	3.73
nocryo	breastred	s3	cISO	i3T3FL	P2	0.79	16.18	2.57
nocryo	breastred	s3	cISO	i3T3FL	P3	0.67	17.30	0.80
nocryo	breastred	s3	TC	IHFL	P1	0.51	16.71	4.86
nocryo	breastred	s3	TC	IHFL	P2	0.63	17.43	3.95
nocryo	breastred	s3	TC	IHFL	P3	0.70	16.98	2.33
nocryo	breastred	s3	TC	i3T3FL	P1	0.77	15.50	3.33
nocryo	breastred	s3	TC	i3T3FL	P2	0.76	16.03	1.80
nocryo	breastred	s3	TC	i3T3FL	P3	0.54	17.24	0.17
nocryo	facelift	s4	rISO	IHFL	P1	0.93	16.75	9.48
nocryo	facelift	s4	rISO	IHFL	P2	1.01	16.15	6.62
nocryo	facelift	s4	rISO	IHFL	P3	0.85	16.30	3.67
nocryo	facelift	s4	rISO	i3T3FL	P1	0.88	14.97	3.10
nocryo	facelift	s4	rISO	i3T3FL	P2	0.79	15.97	1.30
nocryo	facelift	s4	rISO	i3T3FL	P3	0.56	17.38	0.07
nocryo	facelift	s4	cISO	IHFL	P1	0.94	17.02	10.38
nocryo	facelift	s4	cISO	IHFL	P2	0.99	15.80	7.05
nocryo	facelift	s4	cISO	IHFL	P3	0.86	16.29	3.76
nocryo	facelift	s4	cISO	i3T3FL	P1	0.89	14.99	5.23
nocryo	facelift	s4	cISO	i3T3FL	P2	0.82	15.59	2.80
nocryo	facelift	s4	cISO	i3T3FL	P3	0.66	17.51	0.37
nocryo	facelift	s4	TC	IHFL	P1	0.91	16.17	10.10
nocryo	facelift	s4	TC	IHFL	P2	1.05	16.18	5.38
nocryo	facelift	s4	TC	IHFL	P3	0.78	16.43	2.76
nocryo	facelift	s4	TC	i3T3FL	P1	0.85	14.63	2.57
nocryo	facelift	s4	TC	i3T3FL	P2	0.67	16.40	0.37
nocryo	facelift	s4	TC	i3T3FL	P3	0.45	16.92	0.13

This table must be imported into R and converted into a data frame to carry out the statistical analyses of the Supplementary Materials Appendix SA. The variable names are intentionally uncapitalized, simple (no special characters or spaces) and as short as possible to facilitate coding in R. **primo** is a categorical random factor which indicates whether the keratinocytes have been cryopreserved after primoculture or not. **anato** is a categorical random factor which indicates from which anatomical site the keratinocytes were isolated from. **pop** is not used in the statistical models herein and simply designates the keratinocyte populations used in this study. **camp** and **flayer** are both the categorical fixed factors investigated here. They indicate what cAMP inducer type was added to the culture medium and on which feeder layer type the keratinocytes were cultured respectively. **pass** is a categorical fixed factor which indicates on which passage the keratinocytes were. **xdd**, **xms**, and **xhfe** are the three response variable (or proliferative potential proxies) measured in this study. They are mean values of technical replicates and stand for daily population doublings, mean cell size and holoclone-forming efficiency respectively.

Supplementary Materials Figure S1. Annotated R script

```
#______________________________Set Up_________________________________

#Set working directory
setwd("C:/Users/CortezGhioS/Desktop")

#Import file, format data, and create data frame
IData<-read.table("SuppMatTable2.txt",sep="\t",header=T)
primo<-IData$primo
anato<-IData$anato
pop<-IData$pop
camp<-IData$camp
flayer<-IData$flayer
pass<-IData$pass
xdd<-IData$xdd
xms<-IData$xms
xhfe<-IData$xhfe
Data<-data.frame(primo,anato,pop,camp,flayer,pass,xms,xdd,xhfe)
Data
```

```
##     primo     anato pop camp flayer pass   xms  xdd  xhfe
## 1    cryo breastred  s1 rISO   iHFL   P1 15.83 1.10 11.10
## 2    cryo breastred  s1 rISO   iHFL   P2 15.81 1.16  9.67
## 3    cryo breastred  s1 rISO   iHFL   P3 15.92 1.07  7.05
## 4    cryo breastred  s1 rISO i3T3FL   P1 14.32 0.91  2.87
## 5    cryo breastred  s1 rISO i3T3FL   P2 15.32 0.79  3.27
## 6    cryo breastred  s1 rISO i3T3FL   P3 16.49 0.64  0.63
## 7    cryo breastred  s1 cISO   iHFL   P1 15.95 1.06  9.05
## 8    cryo breastred  s1 cISO   iHFL   P2 15.82 1.18  9.24
## 9    cryo breastred  s1 cISO   iHFL   P3 15.92 1.11  7.57
## 10   cryo breastred  s1 cISO i3T3FL   P1 14.23 0.92  3.77
## 11   cryo breastred  s1 cISO i3T3FL   P2 14.89 0.83  5.43
## 12   cryo breastred  s1 cISO i3T3FL   P3 15.95 0.72  1.60
## 13   cryo breastred  s1   TC   iHFL   P1 15.68 0.93  7.81
## 14   cryo breastred  s1   TC   iHFL   P2 15.75 1.00  8.43
## 15   cryo breastred  s1   TC   iHFL   P3 15.89 0.96  6.33
## 16   cryo breastred  s1   TC i3T3FL   P1 14.31 0.85  2.57
## 17   cryo breastred  s1   TC i3T3FL   P2 15.95 0.73  1.80
## 18   cryo breastred  s1   TC i3T3FL   P3 16.11 0.46  0.03
## 19   cryo  facelift  s2 rISO   iHFL   P1 16.49 0.93  4.67
## 20   cryo  facelift  s2 rISO   iHFL   P2 16.24 1.15  5.00
## 21   cryo  facelift  s2 rISO   iHFL   P3 16.39 0.87  1.71
## 22   cryo  facelift  s2 rISO i3T3FL   P1 14.43 0.86  3.53
## 23   cryo  facelift  s2 rISO i3T3FL   P2 17.09 0.38  0.17
## 24   cryo  facelift  s2 rISO i3T3FL   P3 15.40 0.17  0.00
## 25   cryo  facelift  s2 cISO   iHFL   P1 15.69 1.03  6.05
## 26   cryo  facelift  s2 cISO   iHFL   P2 15.98 1.01  2.81
## 27   cryo  facelift  s2 cISO   iHFL   P3 16.51 0.82  1.05
## 28   cryo  facelift  s2 cISO i3T3FL   P1 14.26 0.88  3.43
## 29   cryo  facelift  s2 cISO i3T3FL   P2 17.09 0.43  0.40
## 30   cryo  facelift  s2 cISO i3T3FL   P3 15.00 0.19  0.07
## 31   cryo  facelift  s2   TC   iHFL   P1 15.77 1.55  3.29
## 32   cryo  facelift  s2   TC   iHFL   P2 15.85 0.98  2.71
## 33   cryo  facelift  s2   TC   iHFL   P3 16.55 0.61  0.48
```

```
## 34   cryo  facelift  s2   TC i3T3FL   P1 14.42 0.82  2.60
## 35   cryo  facelift  s2   TC i3T3FL   P2 16.94 0.30  0.00
## 36   cryo  facelift  s2   TC i3T3FL   P3 15.24 0.12  0.00
## 37 nocryo breastred  s3 rISO   iHFL   P1 16.80 0.71  7.67
## 38 nocryo breastred  s3 rISO   iHFL   P2 16.86 0.82  6.43
## 39 nocryo breastred  s3 rISO   iHFL   P3 17.27 0.70  1.71
## 40 nocryo breastred  s3 rISO i3T3FL   P1 16.11 0.68  3.83
## 41 nocryo breastred  s3 rISO i3T3FL   P2 16.58 0.74  1.53
## 42 nocryo breastred  s3 rISO i3T3FL   P3 18.17 0.48  0.33
## 43 nocryo breastred  s3 cISO   iHFL   P1 16.60 0.77  9.00
## 44 nocryo breastred  s3 cISO   iHFL   P2 16.96 0.78  6.29
## 45 nocryo breastred  s3 cISO   iHFL   P3 16.92 0.84  5.33
## 46 nocryo breastred  s3 cISO i3T3FL   P1 15.69 0.76  3.73
## 47 nocryo breastred  s3 cISO i3T3FL   P2 16.18 0.79  2.57
## 48 nocryo breastred  s3 cISO i3T3FL   P3 17.30 0.67  0.80
## 49 nocryo breastred  s3   TC   iHFL   P1 16.71 0.51  4.86
## 50 nocryo breastred  s3   TC   iHFL   P2 17.43 0.63  3.95
## 51 nocryo breastred  s3   TC   iHFL   P3 16.98 0.70  2.33
## 52 nocryo breastred  s3   TC i3T3FL   P1 15.50 0.77  3.33
## 53 nocryo breastred  s3   TC i3T3FL   P2 16.03 0.76  1.80
## 54 nocryo breastred  s3   TC i3T3FL   P3 17.24 0.54  0.17
## 55 nocryo  facelift  s4 rISO   iHFL   P1 16.75 0.93  9.48
## 56 nocryo  facelift  s4 rISO   iHFL   P2 16.15 1.01  6.62
## 57 nocryo  facelift  s4 rISO   iHFL   P3 16.30 0.85  3.67
## 58 nocryo  facelift  s4 rISO i3T3FL   P1 14.97 0.88  3.10
## 59 nocryo  facelift  s4 rISO i3T3FL   P2 15.97 0.79  1.30
## 60 nocryo  facelift  s4 rISO i3T3FL   P3 17.38 0.56  0.07
## 61 nocryo  facelift  s4 cISO   iHFL   P1 17.02 0.94 10.38
## 62 nocryo  facelift  s4 cISO   iHFL   P2 15.80 0.99  7.05
## 63 nocryo  facelift  s4 cISO   iHFL   P3 16.29 0.86  3.76
## 64 nocryo  facelift  s4 cISO i3T3FL   P1 14.99 0.89  5.23
## 65 nocryo  facelift  s4 cISO i3T3FL   P2 15.59 0.82  2.80
## 66 nocryo  facelift  s4 cISO i3T3FL   P3 17.51 0.66  0.37
## 67 nocryo  facelift  s4   TC   iHFL   P1 16.17 0.91 10.10
## 68 nocryo  facelift  s4   TC   iHFL   P2 16.18 1.05  5.38
## 69 nocryo  facelift  s4   TC   iHFL   P3 16.43 0.78  2.76
## 70 nocryo  facelift  s4   TC i3T3FL   P1 14.63 0.85  2.57
## 71 nocryo  facelift  s4   TC i3T3FL   P2 16.40 0.67  0.37
## 72 nocryo  facelift  s4   TC i3T3FL   P3 16.92 0.45  0.13
```

```
#Load necessary packages
library(ARTool) #Non-parametric linear mixed models
```

```
## Loading required package: lme4
```

```
## Loading required package: Matrix
```

```
## Loading required package: Rcpp
```

```
## Loading required package: car
```

```
library(lsmeans) #Post-hoc pairwise comparisons of significant factor
effects (Tukey)
```

```
## Loading required package: estimability
```

```
library(phia) #Post-hoc pairwise comparisons of significant factor
interaction
effects (Holm)
```

```r
#___________________________Daily Doublings______________________________

#ART model fit
ddmod=art(xdd~camp*flayer*pass+(1|primo)+(1|anato),data=Data)
summary(ddmod)
```

```
## Aligned Rank Transform of Factorial Model
##
## Call:
## art(formula = xdd ~ camp * flayer * pass + (1 | primo) + (1 |
##     anato), data = Data)
##
## Column sums of aligned responses (should all be ~0):
##              camp            flayer                pass       camp:flayer
##                 0                 0                   0                 0
##         camp:pass       flayer:pass    camp:flayer:pass
##                 0                 0                   0
##
## F values of ANOVAs on aligned responses not of interest (should all be
~0):
##    Min. 1st Qu.  Median    Mean 3rd Qu.    Max.
##       0       0       0       0       0       0
```

```r
anova(ddmod)
```

```
## Aligned Rank Transform Anova Table (Type III tests)
##
## Response: art(xdd)
##                        F Df Df.res    Pr(>F)
## camp              2.1826  2     52   0.12298
## flayer           50.7902  1     52 3.107e-09 ***
## pass             13.4236  2     52 1.994e-05 ***
## camp:flayer       0.3410  2     52   0.71263
## camp:pass         0.3604  4     52   0.83566
## flayer:pass       4.3351  2     52   0.01814 *
## camp:flayer:pass  0.1000  4     52   0.98197
## ---
## Signif. codes:  0 '***' 0.001 '**' 0.01 '*' 0.05 '.' 0.1 ' ' 1
```

```r
#Post-hoc tests of significant factor effects
lsmeans(artlm(ddmod, "flayer"), pairwise ~ flayer)
```

```
## NOTE: Results may be misleading due to involvement in interactions
```

```
## $lsmeans
##  flayer   lsmean       SE   df lower.CL upper.CL
##  i3T3FL 22.48611 4.357767 1.48 -4.23582 49.20804
##  iHFL   50.51389 4.357767 1.48 23.79196 77.23582
##
## Results are averaged over the levels of: camp, pass
## Confidence level used: 0.95
##
## $contrasts
##  contrast       estimate       SE df t.ratio p.value
##  i3T3FL - iHFL -28.02778 3.932773 52  -7.127  <.0001
##
## Results are averaged over the levels of: camp, pass
```

```
plot(xdd~flayer)
```

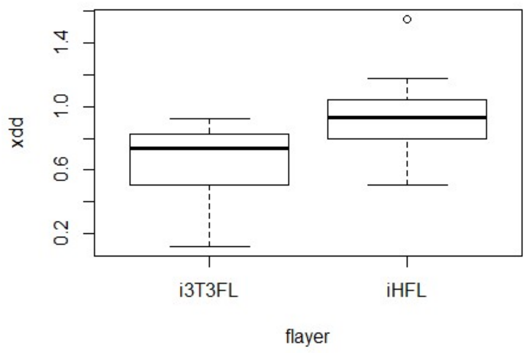


```
lsmeans(artlm(ddmod, "pass"), pairwise ~ pass)
```

```
## NOTE: Results may be misleading due to involvement in interactions
```

```
## $lsmeans
##  pass   lsmean       SE   df  lower.CL upper.CL
##  P1   47.20833 5.541129 2.09 24.311894 70.10477
##  P2   41.95833 5.541129 2.09 19.061894 64.85477
##  P3   20.33333 5.541129 2.09 -2.563106 43.22977
##
## Results are averaged over the levels of: camp, flayer
## Confidence level used: 0.95
##
## $contrasts
##  contrast estimate       SE df t.ratio p.value
##  P1 - P2     5.250 5.498374 52   0.955  0.6084
##  P1 - P3    26.875 5.498374 52   4.888  <.0001
##  P2 - P3    21.625 5.498374 52   3.933  0.0007
##
## Results are averaged over the levels of: camp, flayer
## P value adjustment: tukey method for comparing a family of 3 estimates
```

```
plot(xdd~pass)
```

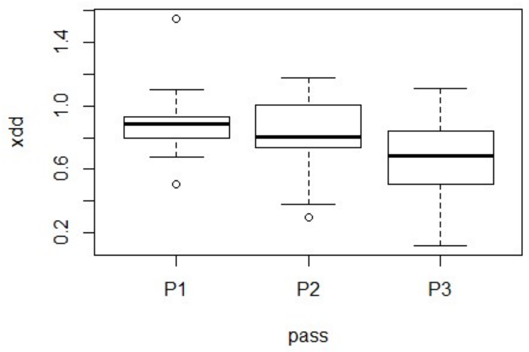


```
#Post-hoc test of the significant factor interaction effect
testInteractions(artlm(ddmod, "flayer:pass"), pairwise=c("flayer",
"pass"), adjustment="holm")
```

```
## Chisq Test:
## P-value adjustment method: holm
##                          Value Df  Chisq Pr(>Chisq)
## i3T3FL-iHFL : P1-P2 30.542  1 6.1136    0.02683 *
## i3T3FL-iHFL : P1-P3 32.375  1 6.8696    0.02630 *
## i3T3FL-iHFL : P2-P3  1.833  1 0.0220    0.88201
## ---
## Signif. codes:  0 '***' 0.001 '**' 0.01 '*' 0.05 '.' 0.1 ' ' 1
```

```
interaction.plot(pass, flayer, xdd, fun = mean)
```

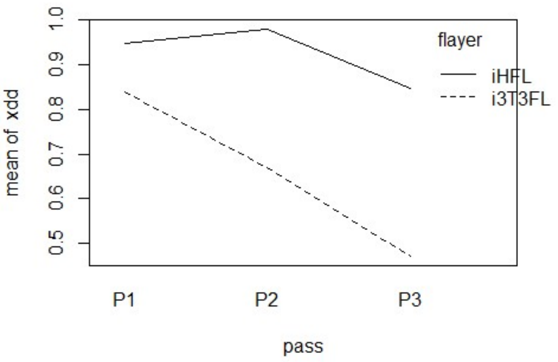


```
#_________________________Mean cell size____________________________

#ART model fit
msmod=art(xms~camp*flayer*pass+(1|primo)+(1|anato),data=Data)
summary(msmod)
```

```
## Aligned Rank Transform of Factorial Model
##
## Call:
## art(formula = xms ~ camp * flayer * pass + (1 | primo) + (1 |
##     anato), data = Data)
##
## Column sums of aligned responses (should all be ~0):
##             camp           flayer             pass      camp:flayer
##                0                0                0                0
##        camp:pass      flayer:pass camp:flayer:pass
##                0                0                0
##
## F values of ANOVAs on aligned responses not of interest (should all be
~0):
##    Min. 1st Qu.  Median    Mean 3rd Qu.    Max.
##       0       0       0       0       0       0
```

```
anova(msmod)
```

```
## Warning in optwrap(optimizer, devfun, getStart(start, rho$lower,
rho$pp), :
## convergence code 3 from bobyqa: bobyqa -- a trust region step failed
to
## reduce q
```

```
## Aligned Rank Transform Anova Table (Type III tests)
##
```

```
## Response: art(xms)
##                         F Df Df.res     Pr(>F)
## camp               0.9105  2     52   0.408631
## flayer            10.8800  1     52   0.001758 **
## pass              18.4371  2     52 8.870e-07 ***
## camp:flayer        0.1734  2     52   0.841247
## camp:pass          0.4121  4     52   0.799101
## flayer:pass       15.2170  2     52 6.271e-06 ***
## camp:flayer:pass   0.3438  4     52   0.847101
## ---
## Signif. codes:  0 '***' 0.001 '**' 0.01 '*' 0.05 '.' 0.1 ' ' 1
```

```
#Post-hoc tests of significant factor effects
lsmeans(artlm(msmod, "flayer"), pairwise ~ flayer)
```

```
## NOTE: Results may be misleading due to involvement in interactions
```

```
## $lsmeans
##  flayer lsmean       SE   df    lower.CL upper.CL
##  i3T3FL   29.5 12.02212 1.06 -103.09475 162.0947
##  iHFL     43.5 12.02212 1.06  -89.09475 176.0947
##
## Results are averaged over the levels of: camp, pass
## Confidence level used: 0.95
##
## $contrasts
##  contrast      estimate      SE df t.ratio p.value
##  i3T3FL - iHFL      -14 4.24437 52  -3.298  0.0018
##
## Results are averaged over the levels of: camp, pass
```

```
plot(xms~flayer)
```

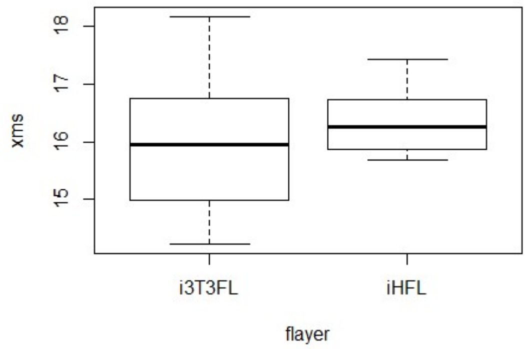


```
lsmeans(artlm(msmod, "pass"), pairwise ~ pass)
```

```
## NOTE: Results may be misleading due to involvement in interactions
```

```
## $lsmeans
##  pass   lsmean       SE   df  lower.CL upper.CL
##  P1   21.08333 10.85968 1.13 -84.31321 126.4799
##  P2   39.95833 10.85968 1.13 -65.43821 145.3549
##  P3   48.45833 10.85968 1.13 -56.93821 153.8549
##
## Results are averaged over the levels of: camp, flayer
## Confidence level used: 0.95
```

```
##
## $contrasts
##  contrast estimate       SE df t.ratio p.value
##  P1 - P2   -18.875 4.614748 52  -4.090  0.0004
##  P1 - P3   -27.375 4.614748 52  -5.932  <.0001
##  P2 - P3    -8.500 4.614748 52  -1.842  0.1662
##
## Results are averaged over the levels of: camp, flayer
## P value adjustment: tukey method for comparing a family of 3 estimates
```

```
plot(xms~pass)
```

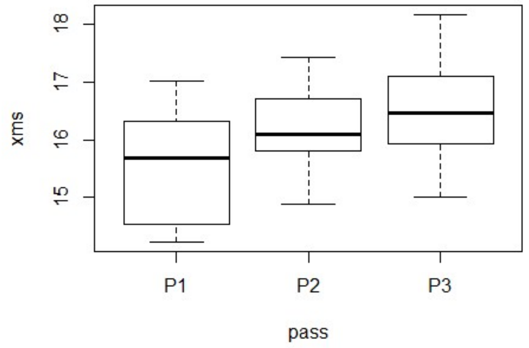


```
#Post-hoc test of the significant factor interaction effect
testInteractions(artlm(msmod, "flayer:pass"), pairwise=c("flayer",
"pass"), adjustment="holm")
```

```
## Chisq Test:
## P-value adjustment method: holm
##                              Value Df   Chisq Pr(>Chisq)
## i3T3FL-iHFL : P1-P2 -43.333  1 21.3390  7.696e-06 ***
## i3T3FL-iHFL : P1-P3 -46.167  1 24.2207  2.577e-06 ***
## i3T3FL-iHFL : P2-P3  -2.833  1  0.0912     0.7626
## ---
## Signif. codes:  0 '***' 0.001 '**' 0.01 '*' 0.05 '.' 0.1 ' ' 1
```

```
interaction.plot(pass, flayer, xms, fun = mean)
```

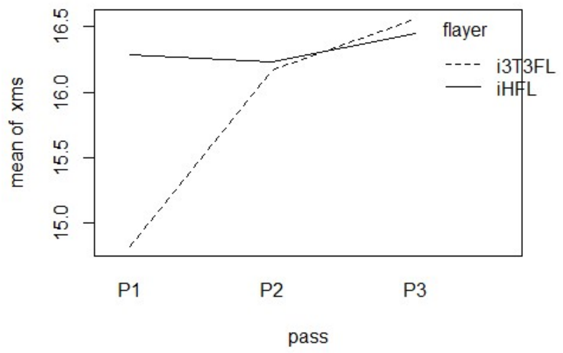


```
#_____________________Holoclone forming efficiency___________________

#ART model fit
```

```
hfemod=art(xhfe~camp*flayer*pass+(1|primo)+(1|anato),data=Data)
summary(hfemod)
```

```
## Aligned Rank Transform of Factorial Model
##
## Call:
## art(formula = xhfe ~ camp * flayer * pass + (1 | primo) + (1 |
##     anato), data = Data)
##
## Column sums of aligned responses (should all be ~0):
##              camp            flayer              pass       camp:flayer
##                 0                 0                 0                 0
##         camp:pass       flayer:pass  camp:flayer:pass
##                 0                 0                 0
##
## F values of ANOVAs on aligned responses not of interest (should all be
~0):
##    Min. 1st Qu.  Median    Mean 3rd Qu.    Max.
##       0       0       0       0       0       0
```

```
anova(hfemod)
```

```
## Aligned Rank Transform Anova Table (Type III tests)
##
## Response: art(xhfe)
##                          F Df Df.res     Pr(>F)
## camp                6.6034  2     52   0.002783 **
## flayer            139.0165  1     52 2.607e-16 ***
## pass               27.9446  2     52 5.737e-09 ***
## camp:flayer         1.1803  2     52   0.315295
## camp:pass           0.3358  4     52   0.852609
## flayer:pass         2.2368  2     52   0.116976
## camp:flayer:pass    0.4339  4     52   0.783477
## ---
## Signif. codes:  0 '***' 0.001 '**' 0.01 '*' 0.05 '.' 0.1 ' ' 1
```

```
#Post-hoc tests of significant factor effects
lsmeans(artlm(hfemod, "camp"), pairwise ~ camp)
```

```
## NOTE: Results may be misleading due to involvement in interactions
```

```
## $lsmeans
##  camp   lsmean       SE   df  lower.CL upper.CL
##  cISO 46.20833 9.133385 1.31 -21.54423 113.9609
##  rISO 37.58333 9.133385 1.31 -30.16923 105.3359
##  TC   25.70833 9.133385 1.31 -42.04423  93.4609
##
## Results are averaged over the levels of: flayer, pass
## Confidence level used: 0.95
##
## $contrasts
##  contrast    estimate       SE df t.ratio p.value
##  cISO - rISO    8.625 5.664554 52   1.523  0.2888
##  cISO - TC     20.500 5.664554 52   3.619  0.0019
##  rISO - TC     11.875 5.664554 52   2.096  0.1004
##
## Results are averaged over the levels of: flayer, pass
## P value adjustment: tukey method for comparing a family of 3 estimates
```

```r
plot(xhfe~camp)
```

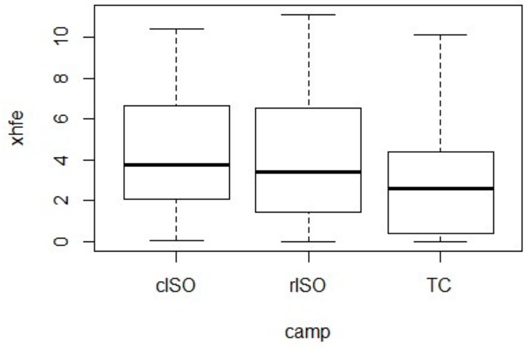


```r
lsmeans(artlm(hfemod, "flayer"), pairwise ~ flayer)
```

```
## NOTE: Results may be misleading due to involvement in interactions
```

```
## $lsmeans
##  flayer   lsmean       SE   df   lower.CL  upper.CL
##  i3T3FL 19.55556 5.980786 1.12 -39.430923  78.54203
##  iHFL   53.44444 5.980786 1.12  -5.542034 112.43092
##
## Results are averaged over the levels of: camp, pass
## Confidence level used: 0.95
##
## $contrasts
##  contrast       estimate       SE df t.ratio p.value
##  i3T3FL - iHFL -33.88889 2.874247 52 -11.791  <.0001
##
## Results are averaged over the levels of: camp, pass
```

```r
plot(xhfe~flayer)
```

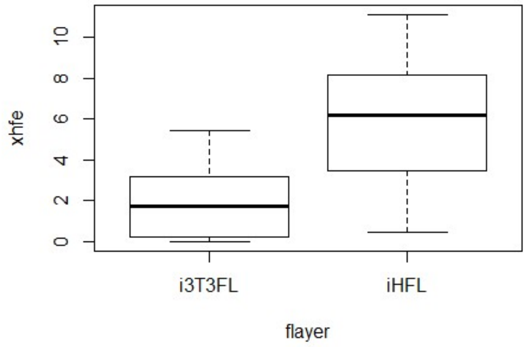


```r
lsmeans(artlm(hfemod, "pass"), pairwise ~ pass)
```

```
## NOTE: Results may be misleading due to involvement in interactions
```

```
## $lsmeans
##  pass   lsmean       SE   df   lower.CL  upper.CL
##  P1   53.04167 7.609867 1.29  -5.167541 111.25087
##  P2   37.50000 7.609867 1.29 -20.709208  95.70921
##  P3   18.95833 7.609867 1.29 -39.250875  77.16754
```

```
##
## Results are averaged over the levels of: camp, flayer
## Confidence level used: 0.95
##
## $contrasts
##  contrast estimate       SE df t.ratio p.value
##  P1 - P2   15.54167 4.564977 52   3.405  0.0036
##  P1 - P3   34.08333 4.564977 52   7.466  <.0001
##  P2 - P3   18.54167 4.564977 52   4.062  0.0005
##
## Results are averaged over the levels of: camp, flayer
## P value adjustment: tukey method for comparing a family of 3 estimates
```

```
plot(xhfe~pass)
```

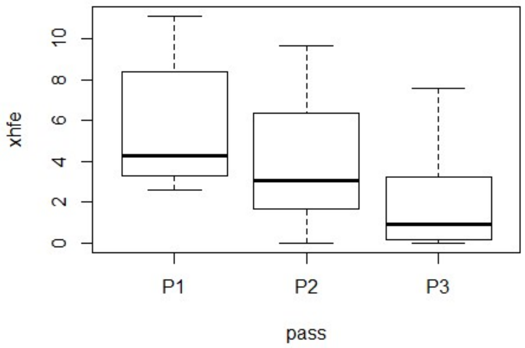

Supplement: Supplementary file 1 [file ijms-19-02174-s001.pdf]
